# Design of a Dual Agonist of Exendin-4 and FGF21 as a Potential Treatment for Type 2 Diabetes Mellitus and Obesity

**DOI:** 10.5812/ijpr-131015

**Published:** 2023-08-09

**Authors:** Changzhen Zhang, Guosheng Gao, Yafeng Li, Jingjing Ying, Jianhui Li, Supei Hu

**Affiliations:** 1 Department of Pharmacy, Ningbo No.2 Hospital, Ningbo, China; 2 Department of Clinical Laboratory, Ningbo No.2 Hospital, Ningbo, China; 3 Department of Pharmacology, Duchuangsanzhong Biotech Co., Ltd., Jiaxing, China; 4 Department of Endocrinology, Ningbo No.2 Hospital, Ningbo, China; 5 Department of Science and Education, Ningbo No.2 Hospital, Ningbo, China

**Keywords:** Fibroblast Growth Factor 21, Exendin-4, Designed Ankyrin Repeat Proteins, Diabetes, Obesity

## Abstract

**Background:**

Fibroblast growth factor 21 (FGF21) is a metabolic, endocrine hormone regulating insulin sensitivity, energy expenditure, and lipid metabolism. It has significant potential as a therapeutic drug for treating type 2 diabetes and obesity. However, the clinical efficacy of FGF21 analogs is limited due to their instability and short half-life. Glucagon-like peptide 1 (GLP-1) receptor agonists have been recognized as effective medications for type 2 diabetes mellitus and obesity over the past two decades.

**Methods:**

This study designed a new long-acting dual-agonist, exendin-4/FGF21, utilizing albumin-binding-designed ankyrin repeat proteins (DARPins) as carriers. The purified fusion proteins were subcutaneously injected into mice for pharmacokinetic and biological activity studies.

**Results:**

Ex-DARP-FGF21 had a high binding affinity for human serum albumin (HSA) in vitro and a prolonged half-life of 27.6 hours in vivo. Bioactivity results reveal that Ex-DARP-FGF21 significantly reduced blood glucose levels in healthy mice. Moreover, compared to Ex-DARP alone, the Ex-DARP-FGF21 dual agonist displayed enhanced blood glucose lowering bioactivity and superior body weight management in the diet-induced obesity (DIO) mouse model.

**Conclusions:**

These results indicate that the long-acting dual agonist of exendin-4 and FGF21 holds considerable potential as a treatment for type 2 diabetes mellitus (T2DM) and obesity in the future.

## 1. Background

Diabetes represents a significant threat to global health as one of the most serious chronic metabolic diseases. In 2021, nearly 537 million people worldwide had diabetes, while around 541 million had impaired glucose tolerance (1). Diabetic patients grapple with elevated blood glucose levels, cardiovascular diseases, and other complications such as obesity, diabetic retinopathy, diabetic foot, and non-alcoholic fatty liver disease (NAFLD) (2). Accounting for over 90% of all global diabetes cases, type 2 diabetes mellitus (T2DM) is largely caused by the body cells' inability to fully respond to insulin, known as insulin resistance (3). Research indicates that roughly 80 - 90% of patients with T2DM are overweight or obese (4). Additionally, weight loss has been proven to yield significant benefits for patients with T2DM (5). Hence, developing reliable hypoglycemic drugs that can also manage cardiovascular risks and body weight is essential.

As a powerful metabolic endocrine hormone, fibroblast growth factor 21 (FGF21) regulates insulin sensitivity, energy expenditure, and lipid metabolism (6, 7). Thus, FGF21 and its analogs have been identified as potentially effective therapeutic drugs for conditions like type 2 diabetes, obesity, and NAFLD (8). Also, FGF21 acts by binding to the β-klotho receptor and fibroblast growth factor receptor 1c (FGFR1c), resulting in a stable FGF21/β-klotho/FGFR complex. Besides, β-klotho, expressed in specific tissues, enhances the interaction between FGFR1c and FGFR21, guiding FGF21 signaling to those specific tissues (9). Rodent models have demonstrated that a single dose of FGF21 can significantly improve insulin sensitivity and decrease blood glucose levels by over 50% (10). The insulin-sensitizing effects of FGF21 are achieved through its direct impact on adipose tissues, as it boosts glucose uptake by white adipocytes (11). In addition, long-term administration of FGF21 has been shown to significantly reduce body weight by increasing energy expenditure, even without a reduction in food intake (12). Recent studies have linked FGF21-mediated weight loss to the central nervous system (CNS) (13), although the exact mechanism is not yet fully understood. However, the clinical application of FGF21 is limited due to its short half-life, susceptibility to protease degradation, and propensity to aggregate (14). Furthermore, FGF21 analogs have demonstrated less clinical efficacy in reducing blood glucose levels and body weight in humans than in rodents (8). Therefore, there is a need to develop more potent and effective FGF21 analog drugs for treating diabetes and obesity.

Glucagon-like peptide 1 (GLP-1) receptor agonists form a group of gastrointestinal peptides that augment glucose-stimulated insulin secretion and suppress glucagon secretion (15, 16). Over the past two decades, various GLP-1 receptor agonists have been approved for treating T2DM and obesity. Exenatide, the first GLP-1 receptor agonist approved by the Food and Drug Administration (FDA) in 2005 for T2DM treatment, is a 39-amino acid peptide secreted by the Gila monster's salivary glands. It shares a 53% sequence homology with human GLP-1. Thanks to its resistance to DPP-4 degradation, exenatide has a longer half-life than natural GLP-1, necessitating a twice-daily administration (17-19). To enhance the pharmacological attributes of GLP-1 receptor agonists, the FDA and the European Medicines Agency (EMA) have approved three strategies for extending their half-lives: (1) fatty acid chain modification (as in liraglutide and semaglutide); (2) sequence mutation to avoid degradation by dipeptidyl peptidase 4; and (3) fusion with human serum albumin (HSA) or IgG-Fc (as in albiglutide and dulaglutide) (20). These modifications serve not only to increase the size of the drug, thereby reducing renal clearance but also to co-opt the recycling of the neonatal Fc receptor (FcRn) via FcRn-Fc or FcRn-albumin interactions (21). By employing these strategies, semaglutide, and dulaglutide have achieved extended half-lives of up to 7 and 5 days in humans, respectively (22, 23).

Designed ankyrin repeat proteins (DARPin) are artificially engineered and selected based on the natural ankyrin protein. They can specifically bind to various proteins (24-26). Each DARPin domain consists of 33 amino acid residues, which include several repeating modules for target binding and N-terminal and C-terminal cap modules to stabilize the structure (27). Also, DARPin displays advantageous biochemical properties for medical applications, such as high thermostability, excellent solubility, robust production in *E. coli*, and impressive tissue penetration (25, 28). Moreover, DARPin targets various epitopes that can be conveniently fused into a bi-specific or tri-specific drug. For instance, MP0250, a DARPin drug candidate currently in phase 2 clinical trials, comprises a vascular endothelial growth factor (VEGF)-binding domain, hepatocyte growth factor (HGF)-binding domain, and HSA binding domain. This tri-specific drug exhibits dual inhibitory activity targeting VEGF and HGF for cancer treatment, and due to HSA binding, it has an exceptionally long half-life of 11 days in patients (29).

## 2. Objectives

In this study, we designed a novel dual agonist to target GLP-1 and FGF21 receptors. The fusion protein was successfully expressed in the *E. coli* system and purified. Our results demonstrated that the administration of Ex-DARP-FGF21 significantly reduced body weight and circulating glucose levels in both normal mice and the diet-induced obesity (DIO) model. Consequently, this dual agonist could become a promising drug candidate for treating diabetes and obesity.

## 3. Methods

### 3.1. Construction of Fusion Proteins

In order to construct a long-acting GLP-1/FGF21 dual-agonist fusion protein, we employed the albumin-binding DARPin moiety derived from MP0250 to connect one or two agonists. The GLP-1 receptor agonist, exendin-4 peptide (GenBank: AAB51130.1), was fused to the N-terminus of DARPin using a (GGGGS)3 flexible linker. This configuration was referred to as Ex-DARP. Furthermore, FGF21 (GenBank: BAA99415.1), featuring L98R and P171A amino acid substitutions, was fused to the C-terminus of DARPin via the same type of flexible linker (GGGGS)3 and designated as DARP-FGF21. To facilitate the acquisition of the free N-terminus of exendin-4, a TEV protease cleavage site was placed at the N-terminal of exendin-4. The DNA sequences encoding the Ex-DARP and Ex-DARP-FGF21 fusion proteins were codon-optimized for *E. coli* expression and synthesized by GENEWIZ (Suzhou, China). The synthesized genes were cleaved using *Nco* I and *Xho* I enzymes and then cloned into a pET-28a(+) plasmid, preserving the N-terminal His-tag for subsequent affinity chromatography.

### 3.2. Protein Expression and Purification

The fusion protein-encoding expression plasmids were transformed into BL21(DE3) cells. A single clone was inoculated into 5 mL of LB medium with 50 μg/mL of kanamycin and incubated overnight at 37°C. This starter culture was then added to 5 L of LB medium and grown at 37°C until the OD600 reached 0.6 - 0.8. Protein expression was induced by adding Isopropyl-β-D-1-thiogalactopyranoside to a final concentration of 0.1 mM, followed by incubation at 20°C for 16 h. The cells were harvested by centrifugation at 5,000 rpm for 15 min and resuspended in lysis buffer (20 mM Tris/HCl, 300 mM NaCl, 20 mM imidazole, pH 8.0). After undergoing high-pressure homogenization (ATS, China) at 4°C, the cell lysates were centrifuged at 15,000 rpm at 4°C for 30 min to remove insoluble precipitates. The resulting supernatant was loaded onto a nickel affinity chromatography column (GE Healthcare, USA) pre-equilibrated with lysis buffer. The column was washed with 10 column volumes of washing buffer (20 mM Tris/HCl, 300 mM NaCl, 40 mM imidazole, pH 8.0). The fusion protein was eluted using elution buffer (20 mM Tris/HCl, 300 mM NaCl, and 200 mM imidazole, pH 8.0) and subsequently dialyzed to remove imidazole (30).

To remove the His-tag, a 25 mL fusion protein solution was incubated with 0.5 mg of TEV protease, 1 mM EDTA, and 1 mM DTT at 25°C for 2 h (31). The protein solution was further purified using a Ni^2+^-NTA column, after which the unbound protein, devoid of His-tag, was collected and dialyzed in PBS. The purity and molecular composition of the final proteins were analyzed using 12.5% SDS-PAGE. The protein concentration was determined using a BCA assay kit (CWBIO, China).

Western blotting was utilized to identify the target protein, capitalizing on the specificity of the antibody-antigen interaction. Initially, the purified fusion proteins were separated by 4 - 20% gradient electrophoresis and transferred onto PVDF membranes. The membrane was blocked with 5% nonfat milk overnight at 4°C to prevent non-specific binding of the antibody to the membrane surface. The membrane was then rinsed with TBST, and the primary antibodies against exendin-4 (Abcam, UK) and HRP-labeled secondary antibody (Solarbio, Beijing, China) were successively incubated with the PVDF membranes for 2 h. Subsequently, the membrane was treated with ECL developing solution, and the protein expression band was visualized using a Tanon 5200 chemiluminescence image system (Tanon, Shanghai, China).

### 3.3. Binding Affinity of Fusion Protein for Human Serum Albumin In Vitro

The binding abilities of Ex-DARP and Ex-DARP-FGF21 to HSA were determined using enzyme-linked immunosorbent assay (ELISA), as described by Tan et al. (31). Briefly, 10 μg of HSA was captured in a coating solution (100 mM carbonate buffer, pH 9.6) and immobilized in a 96-well plate by incubating overnight at 4°C. After washing three times with PBST (PBS + 0.05% tween 20), the immobilized plate was blocked with a reagent (PBS with 10% chicken serum). Then, 100 μL of the fusion protein at various concentrations was added to each well and incubated at room temperature for 2 h. Exendin-4 in fusion proteins was detected using a mouse anti-exendin-4 antibody (Abcam, UK) and HRP-conjugated goat anti-mouse IgG (CWBIO, China). After three washes, TMB substrate (Solarbio, China) was added to detect bound HRP. The reaction was halted using HCl (1 M), and absorbance was measured at 450 nm with a SpectraMax M5 microplate reader (Molecular Devices, USA). The GraphPad Prism program calculated the half-maximal binding concentration or EC50.

### 3.4. Animals and Treatment

Eight-week-old male C57BL/6 mice (approximately 20 g) were obtained from the Academy of Military Medical Sciences and housed under a 12-hour light/dark cycle at 25°C. The mice were given standard food (18% kcal fat) and water ad libitum. For the DIO model, mice were fed a high-fat diet (HFD, 60% kcal fat) for 12 weeks before the study (32). The mice's body weights in the lean and DIO groups were recorded weekly. After 12 weeks of HFD feeding, fasting glucose levels were measured using a glucometer (Sanocare, China). All experiments were conducted according to the guidelines for the care and use of laboratory animals as outlined by the Animal Care Committee of China. All experimental protocols received approval from the Ningbo University Laboratory Animal Center (ethics code: 11384).

### 3.5. Pharmacokinetic Studies in Mice

For the pharmacokinetic studies, purified Ex-DARP-FGF21 with a His-tag was administered subcutaneously to the C57BL/6 mice at a dose of 10 nmol/kg. Approximately 40 μL of blood was collected from the orbital venous plexus at different time points (1, 3, 8, 12, 24, 36, 48, and 72 h). The plasma was separated by centrifugation at 4,000 g for 10 min at 4°C and then diluted with PBS. The plasma concentration of the fusion protein was measured following the method described by Schellenberger et al. (33). Briefly, an anti-exendin-4 mouse monoclonal antibody (Abcam, UK) was diluted with a carbonate-bicarbonate buffer (pH 9.6), immobilized in a 96-well plate, and incubated overnight at 4°C. It was then blocked with a reagent (PBS with 10% chicken serum). After washing three times, standard and plasma samples were added to the wells and incubated at 25°C for 2 h. The bound fusion proteins were detected using a rabbit anti-FGF21 antibody (Abcam, UK) and HRP-conjugated goat anti-rabbit IgG (CWBIO, China). Subsequent procedures were performed as previously described. The concentration of the fusion protein was calculated based on a standard curve. The half-life of the fusion protein was determined using the PKSolver program in Microsoft Excel.

### 3.6. Biological Activities of Fusion Protein In Vivo

Healthy C57BL/6 mice were randomly divided into five groups (4 - 5 mice each) and fasted overnight. Fusion proteins Ex-DARP-FGF21 and Ex-DARP were injected subcutaneously at high doses of 30 nmol/kg and low doses of 10 nmol/kg to determine non-fasting blood glucose levels in normal mice. Blood glucose was measured from the tail vein using a glucometer (Sanocare, China) at various time points after injection (1, 3, 8, 12, 24, 36, 48, and 72 h) (31). The duration of the hypoglycemic effect in normal mice or the DIO model was determined based on a P value below 0.05 compared to the PBS group.

For the Oral Glucose Tolerance Test (OGTT), overnight-fasted C57BL/6 mice were randomly divided into three groups and injected with fusion proteins at 25 nmol/kg. Then, 2 g/kg glucose was administered orally after the fusion protein injection. Blood glucose levels were measured using a glucometer (Sanocare, China) at - 30, 0, 15, 30, 60, and 120 min (34).

To study the long-term therapeutic effects of fusion proteins, overnight-fasted DIO mice were randomly assigned to five groups (4 - 5 mice per group), and fusion proteins were injected subcutaneously at a dose of 25 nmol/kg every three days for 30 days. Blood glucose levels were measured from the tail vein before each injection using a glucometer. Anti-obesity effects were measured by monitoring food intake and body weight throughout the experiment. Food intake and cumulative food consumption were measured during the Ex-DARP-FGF21 and Ex-DARP treatment periods for the DIO mice. Mice were fed a consistent amount of food (approximately 80 g), and food residue was measured every two days to calculate daily and cumulative food intake. Reduction percentages in body weights were calculated using the formula: Reduction percentages = (initial weight - current weight)/initial weight.

### 3.7. Statistical Analysis

The data are presented as the mean ± standard error and analyzed using GraphPad Prism 8.01 (GraphPad Software, USA). A one-way analysis of variance followed by Tukey's test was used for multiple comparisons. Differences were considered statistically significant at a P-value < 0.05.

## 4. Results

### 4.1. Design and Purification of Ex-DARPEx-DARP-FGF21 Fusion Protein

**Figure 1.**
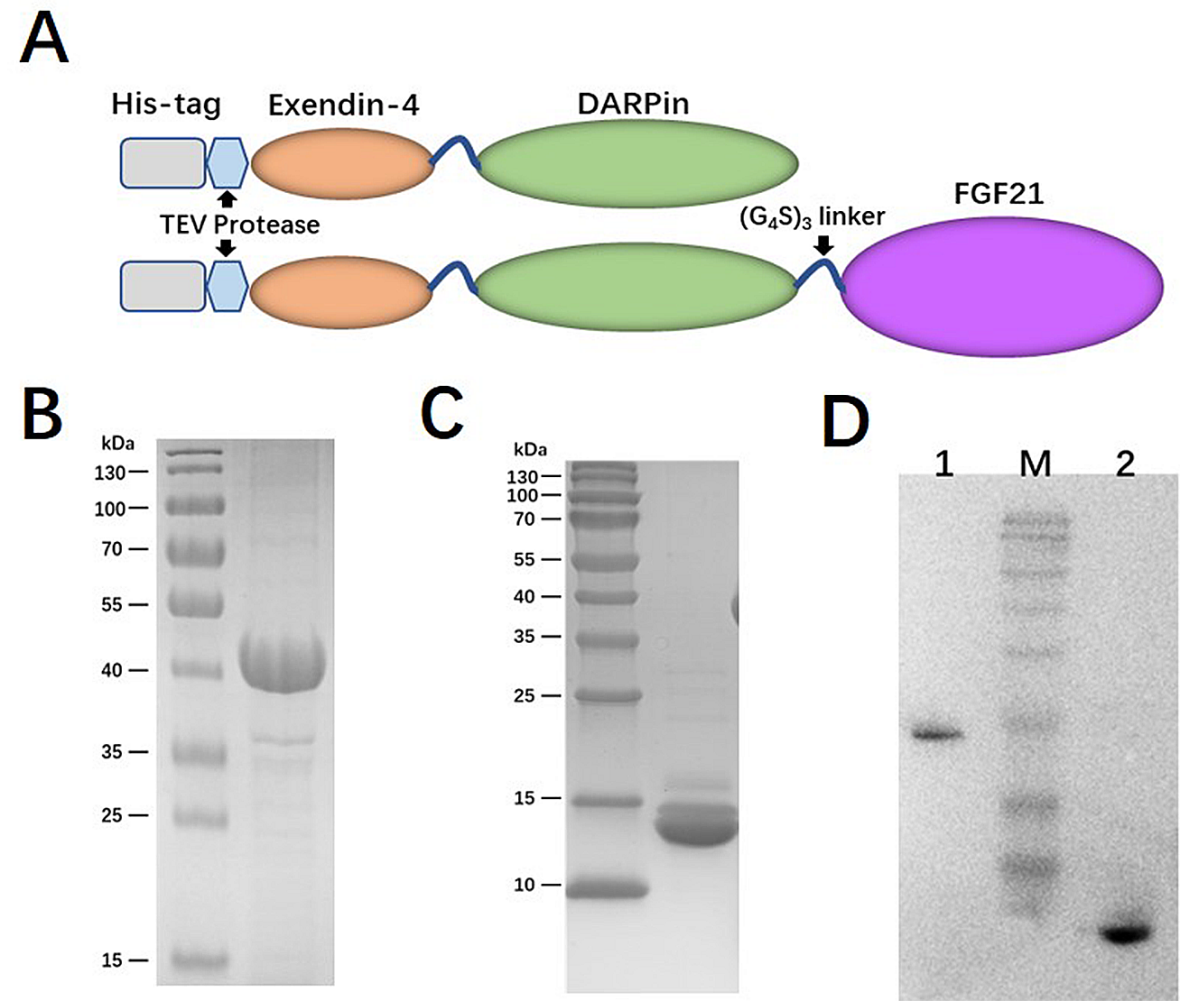
Schematic representation and purification of Ex-DARP and Ex-DARPEx-DARP-FGF21 fusion proteins. A, schematic representation showing the construction of fusion proteins; B, SDS-PAGE analysis of purified Ex-DARP-FGF21; and C, Ex-DARP after affinity chromatography; D, western blot analysis of purified fusion proteins. Lane 1: Purified Ex-DARP-FGF21 fusion protein; lane 2: Purified Ex-DARP fusion protein.

Given the crucial role of receptor binding, a dual agonist was designed using a head-to-tail model (Ex-DARP-FGF21). An exendin-4 peptide was fused to the N-terminus of DARPin, resulting in a free N-terminus of exendin-4 after digestion by the TEV protease, thus creating Ex-DARP. Also, FGF21 was positioned at the C-terminus of the fusion protein, enabling the receptor β-klotho to bind to the C-terminus of FGF21 (7) (Figure 1A). As known, DARPin acts as a carrier to extend the half-life of dual agonist fusion proteins through non-covalent binding to albumin in the blood. To prevent the degradation and aggregation of FGF21, two-point mutations were introduced, as previously reported (14, 35). A flexible linker (GGGGS)3 was inserted between exendin-4, DARPin, and FGF21, improving fusion protein refolding (36). The designed sequence was synthesized and cloned into pET28a(+) to preserve the His-tag at the N-terminus of the fusion protein. The expression of Ex-DARP and Ex-DARP-FGF21 fusion proteins was regulated by the T7 promotor and lac operator in pET vectors, and the production of the target protein was induced by lactose or IPTG by binding to the repressor protein. Both Ex-DARP and Ex-DARP-FGF21 fusion proteins were successfully expressed in the BL21(DE3) strain under induction conditions at 20°C and 0.1 mM IPTG. For many proteins, expression at 20°C not only helps to prevent misfolding that can occur above 30°C but also lessens the negative impacts of low temperature (e.g., 16°C) on protein expression. Both His-tagged fusion proteins were purified using a Ni^2+^-NTA column and then digested with TEV protease to remove the His-tag. High-purity Ex-DARP-FGF21 and Ex-DARP were obtained, yielding 53 mg and 47 mg, respectively, after further purification with a second Ni^2+^-NTA column. The SDS-PAGE and WB analysis revealed the target band with a molecular mass of 39 kDa and 14 kDa, corresponding to the theoretical value of the Ex-DARP-FGF21 and Ex-DARP fusion protein (Figure 1B, C, and D). However, an additional band was also found in Figure 1C, in line with the results of previous studies on other DARPin fusion proteins. For instance, Tan et al. designed a fusion protein, GLP-DARPin, and observed two bands on SDS-PAGE. Both Q ion exchange and size-exclusion chromatography displayed monomeric peaks without signs of aggregation or multimerization. The extra band could be attributed to the incomplete unfolding of DARPin domains on SDS-PAGE due to their high thermodynamic stability (37). The concentrations of the fusion proteins were determined using a BCA assay kit (CWBIO, China).

### 4.2. Ex-DARP-FGF21 High Binding Affinity for HSA In Vitro

**Figure 2.**
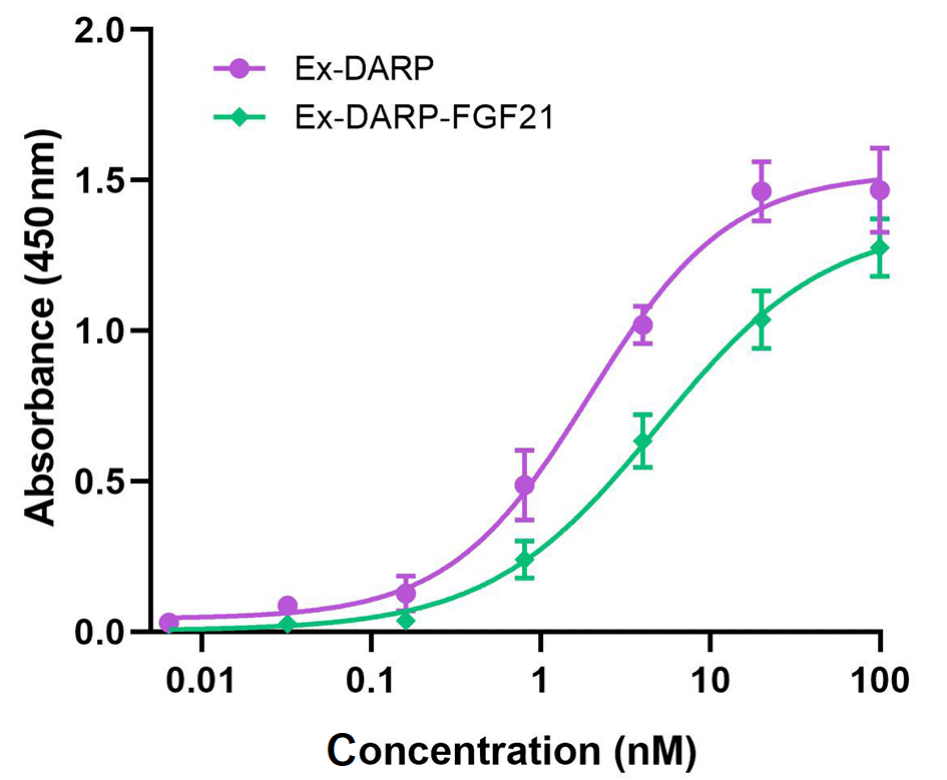
The binding affinities of designed ankyrin repeat protein (DARPin) fusion proteins to human serum albumin (HSA) were analyzed by enzyme-linked immunosorbent assay (ELISA) in vitro. The fusion proteins at diverse doses were captured by immobilized HSA, and the anti-exendin-4 antibody was used to quantify the target fusion proteins.

The binding affinities of Ex-DARP-FGF21 fusion proteins to HSA were determined by ELISA, as described by Tan et al. (31). Briefly, HSA was immobilized on the surface of a 96-well plate to capture fusion proteins containing DARPin, and an anti-exendin antibody was used to detect the bound fusion protein. As illustrated in Figure 2, both Ex-DARP-FGF21 and Ex-DARP were efficaciously bound to HSA with half-maximal binding concentrations of 4.5 nM and 1.9 nM, respectively. These data indicate that both Ex-DARP-FGF21 and Ex-DARP maintained their ability to bind to HSA.

### 4.3. Pharmacokinetics Characterization of Ex-DARP-FGF21 in Mice

**Figure 3.**
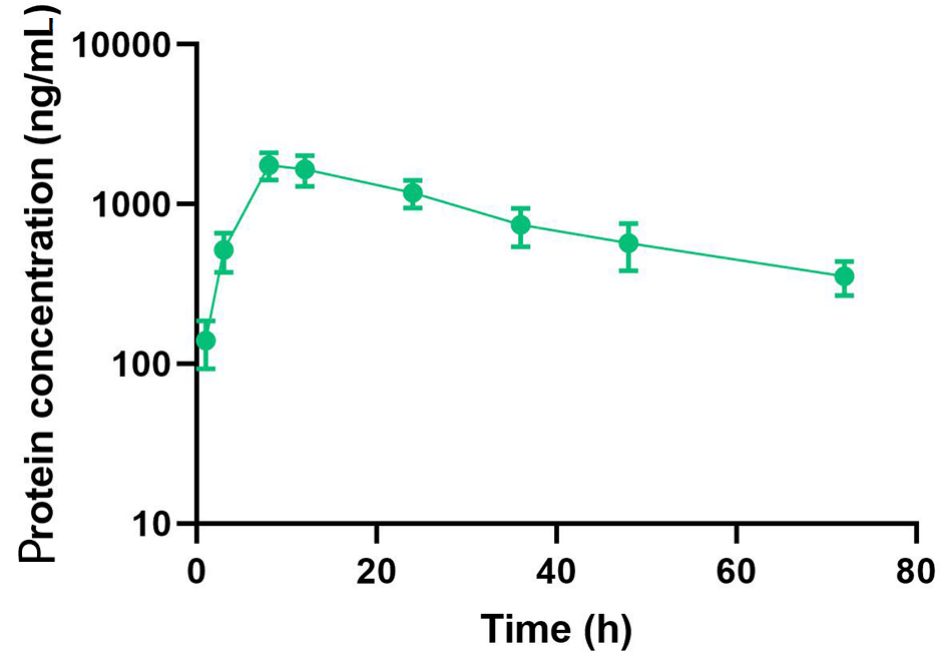
Half-life Determination of Ex-DARPEx-DARP-FGF21 in C57BL/6 Mice. Ex-DARPEx-DARP-FGF21 fusion proteins were subcutaneously injected into C57BL/6 mice (n = 3) at 10 nmol/kg. The plasma concentrations of Ex-DARPEx-DARP-FGF21 proteins at different time points were determined using enzyme-linked immunosorbent assay (ELISA).

The pharmacokinetic properties of the compound were evaluated in C57BL/6 mice following a single subcutaneous injection at a dosage of 10 nmol/kg. The concentration of the dual agonist in the blood was quantified using an ELISA method, employing an anti-exendin-4 antibody as the capture antibody and an anti-FGF21 antibody as the detection antibody. The study results demonstrated that the half-life of Ex-DARP-FGF21 was 27.6 ± 3.2 hours (Figure 3). These data suggest that the designed dual agonist incorporating DARPin displays significant stability in circulation.

### 4.4. Effect of Ex-DARP-FGF21 Dual Agonist on Blood Glucose in Normal Mice

**Figure 4.**
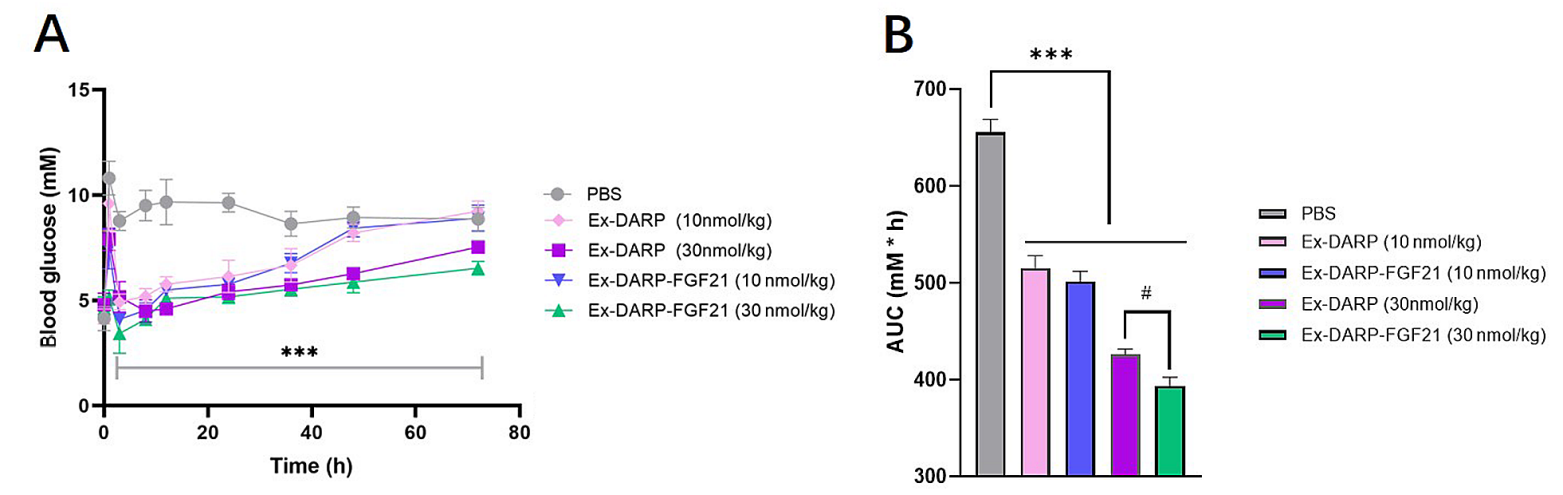
The hypoglycemic effects of Ex-DARP and Ex-DARPEx-DARP-FGF21 in normal mice. The non-fasting blood glucose (A); and calculated area under curve (AUC) values (B) were determined following the subcutaneous injection of Ex-DARP-FGF21 and Ex-DARP at 10 nmol/kg and 30 nmol/kg. C57BL/6 mice (n = 4) were fasted overnight.at different time points. Results are presented as the mean ± standard error * P < 0.05, ** P < 0.01, *** P < 0.001 (vs. PBS group), # P < 0.05 for Ex-DARP (30nmol/kg) group versus Ex-DARP-FGF21 (30 nmol/kg) group.

In order to evaluate the efficacy of the dual agonist in vivo, we examined non-fasting glucose levels. Mice were administered a single subcutaneous injection of Ex-DARP-FGF21 at two doses - a low dose of 10 nmol/kg and a high dose of 30 nmol/kg. As demonstrated in Figure 4, both Ex-DARP-FGF21 and Ex-DARP notably decreased non-fasting blood glucose levels from above 8.0 mM to below 5.0 mM compared to the vehicle control (P < 0.0001). The glucose-lowering effect of Ex-DARP-FGF21 persisted for at least 36 hours at the lower dose and 72 hours at the higher dose. Furthermore, the blood glucose level of Ex-DARP-FGF21 was lower than that of Ex-DARP within the first 2 hours following feeding and injection of the fusion protein. Both the Ex-DARP-FGF21 and Ex-DARP treatments markedly decreased the glucose area under curve (AUC) relative to the vehicle (P < 0.05).

**Figure 5.**
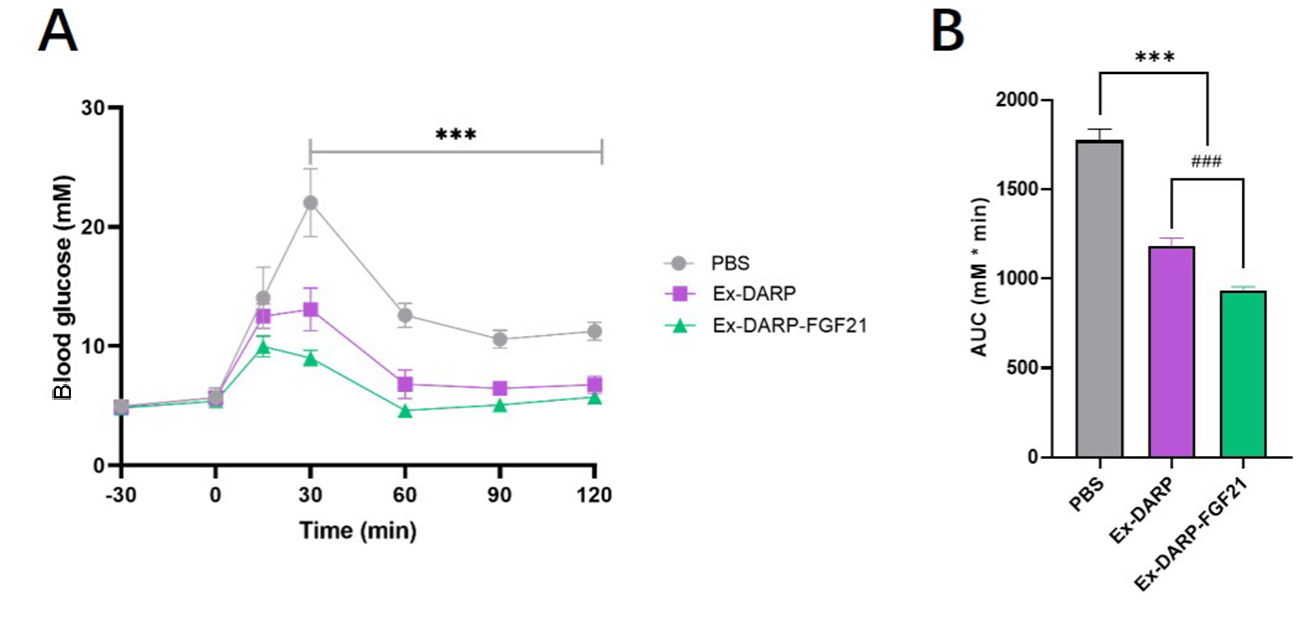
Oral glucose tolerance test in normal C57BL/6 mice. Blood glucose levels (A); and calculated area under curve (AUC) (B) were measured after protein injection in mice. Data are presented as the mean ± standard error, * P < 0.05, ** P < 0.01, *** P < 0.001 (vs. PBS group). ### P < 0.001 (Ex-DARP group vs. Ex-DARP-FGF21 group).

An OGTT (2.0 g/kg) was conducted following overnight fasting. The results indicated that both Ex-DARP-FGF21 and Ex-DARP fusion proteins significantly reduced blood glucose levels between 30 and 120 minutes at a dose of 25 nmol/kg when compared to the PBS group (P < 0.0001) (Figure 5A). Moreover, analysis of the calculated AUC revealed that Ex-DARP-FGF21 exhibited stronger glucose-lowering bioactivity than Ex-DARP (P < 0.0059) (Figure 5B). These data suggest potential synergistic action of the GLP-1 analog and FGF21 for glycemic control.

### 4.5. Effect of Dual Agonist Fusion Protein on Glycemia and Body Weight in Diet-induced Obesity Mice Model

**Figure 6.**
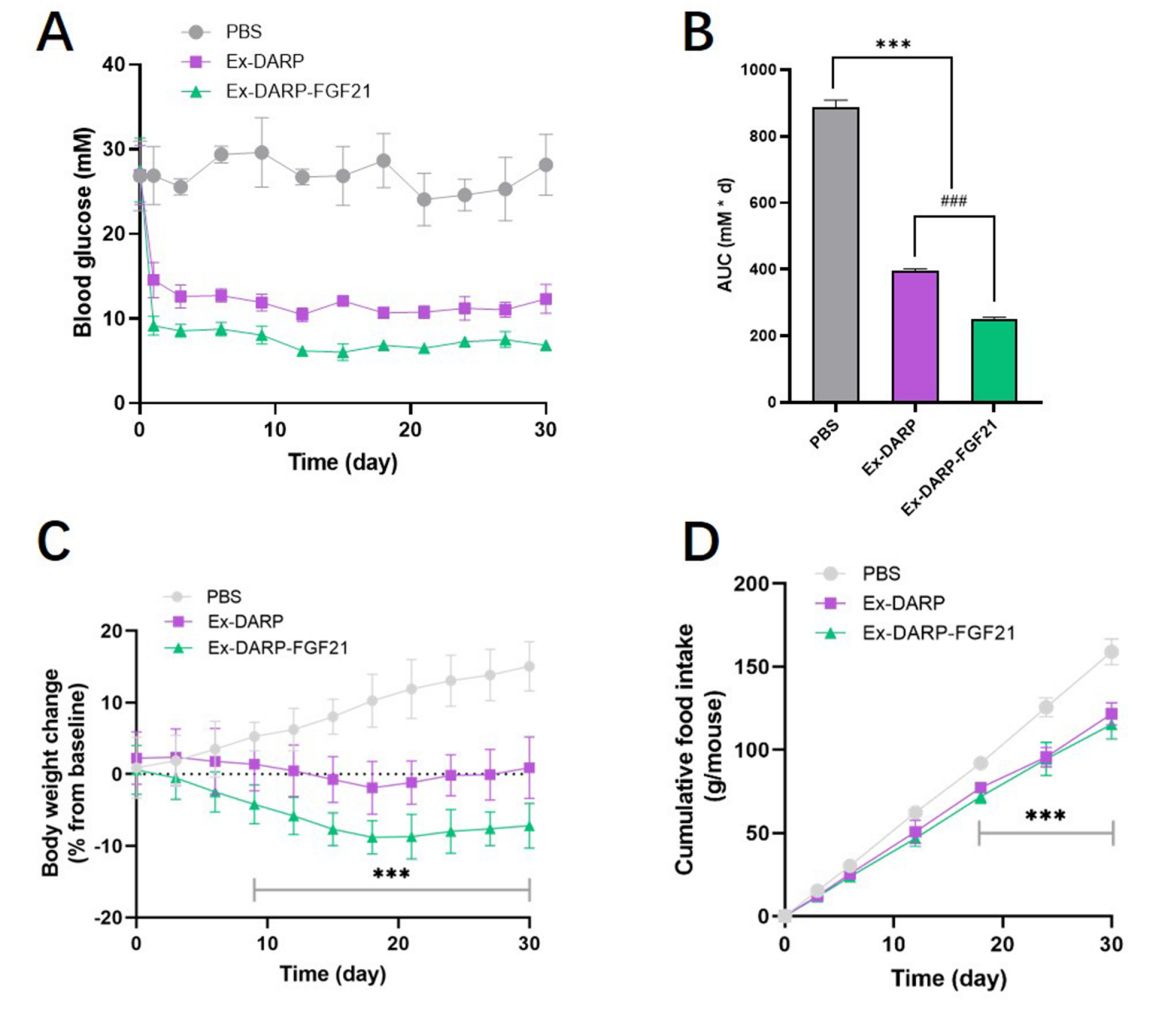
Long-term effects of designed ankyrin repeat proteins (DARPin) fusion proteins on diet-induced obesity (DIO) mice. Blood glucose levels (A); calculated AUC (B); body weight (C); and cumulative food consumption (D) were determined in DIO mice after treatment with 25 nmol/kg DARPin fusion proteins every three days for 30 days. Data are presented as the mean ± standard error, * P < 0.05, ** P < 0.01, *** P < 0.001 (vs. PBS group). ### P < 0.001 (Ex-DARP group vs. Ex-DARP-FGF21 group).

This study further explored the potential application of Ex-DARP-FGF21 and Ex-DARP in treating T2DM by subcutaneously administering these compounds to DIO mice at 30 nmol/kg once every three days for 30 consecutive days. We monitored blood glucose levels and body weights every three days before each injection. As depicted in Figure 6A, both Ex-DARP-FGF21, and Ex-DARP maintained their glucose control effects for at least 28 days (P < 0.0001). Notably, Ex-DARP-FGF21 demonstrated superior bioactivity to Ex-DARP at a high dose (30 nmol/kg), which was statistically significantly different (P < 0.0001) (Figure 6B). These results suggest a robust impact of the dual agonists on glycemic control.

Considering the inhibitory effect of GLP-1 analogs on food intake and the influence of FGF21 on energy expenditure, we postulated that a dual agonist targeting both GLP-1 and FGF21 receptors might synergistically affect body weight control. To test this hypothesis, we measured food intake and body weight in DIO mice. As shown in Figure 6C, Ex-DARP-FGF21 prompted more significant weight loss than PBS, with reductions averaging 22.2 ± 3.7%. Throughout the 30-day period, the body weight of the Ex-DARP-FGF21 and Ex-DARP groups was consistently and steadily maintained at a lower level, a trend likely driven by the inhibition of food intake from day 3 to day 30 (Figure 6D). Interestingly, there was no significant difference between the cumulative food intakes of the Ex-DARP and Ex-DARP-FGF21 groups. This finding suggests that the weight control effect of Ex-DARP-FGF21 may be mediated by factors other than just reduced food intake. These findings suggest that Ex-DARP-FGF21 enhances both glucose-lowering and weight-loss effects throughout the treatment period.

## 5. Discussion

Fibroblast growth factor 21 has been suggested as a promising new drug for treating diabetes and obesity thanks to its multiple metabolic-regulating functions, such as enhancing insulin sensitivity and increasing energy expenditure (7). Fibroblast growth factor 21 treatment has been shown to reduce blood glucose levels by increasing glucose uptake in adipose tissue. However, the clinical application of FGF21 is constrained by its short half-life, susceptibility to protease degradation, and tendency to aggregate. Several strategies have been documented to enhance the stability of FGF21. Two-point mutations (L98R and P171G) can effectively prevent degradation and aggregation without impacting receptor binding affinity (14). In contrast to FGF21, GLP-1 analogs can stimulate insulin secretion and inhibit glucagon secretion in a glucose-dependent manner, thus lowering blood glucose levels. Additionally, GLP-1 analogs delay gastric emptying and suppress food intake, leading to weight loss (16). Furthermore, the inhibition of gluconeogenesis in hepatocytes by GLP-1 analogs is mediated by increased circulating FGF21 levels (38). Therefore, we hypothesized that the synergistic function of GLP-1 and FGF21 could enhance the efficacy of diabetes and obesity treatments.

In this study, we designed a novel dual agonist targeting the GLP-1 and FGF21 receptors, using the albumin-binding DARPin domain as a carrier. The DARPin domain was employed as a carrier to prolong the half-life in blood via albumin binding. Initially, *E. coli* was utilized as the host for heterologous expression, and the target protein was purified for subsequent pharmacological evaluation. Basic pharmacological evaluation involves drug activity and drug metabolism testing. For this research, we conducted non-fasting blood glucose control tests, oral glucose tolerance tests, and hypoglycemic activity tests in DIO mice to verify the drug's blood glucose control capability. Concurrently, given that both GLP-1 and FGF21 possess body weight reduction activity, we also performed experiments on food intake and weight control. For the drug metabolism study, we determined the half-life of drug metabolism in vivo. Additionally, in vitro experiments confirmed the protein's albumin binding ability, shedding light on one reason for the extension of its half-life.

The exendin-4 peptide and FGF21 were positioned at the N-terminal and C-terminal of DARPin, respectively. A flexible peptide (GGGGS)3 was used to interconnect each module. This "head-to-tail" fusion protein design was based on four considerations. Firstly, GLP-1 and FGF21 necessitate exposed N-terminus and C-terminus, respectively, for receptor binding. Similar structures featuring different carrier domains (Fc or elastin-like polypeptide (ELP)) have been reported (39, 40). Secondly, the DARPin domain can bind non-covalently to albumin in vivo, forming the Ex-DARP-FGF-HSA complex with a large molecular size. This prolongs the dual agonist's effect due to slow renal clearance and FcRn-mediated recycling. Thirdly, the DARPin domain exhibits unique biochemical properties beneficial for drug development, including high thermostability, solubility, production in *E. coli*, and tissue penetration (24, 25, 29). Finally, the flexible linker provides the necessary spatial distance between the domains, enhancing binding to respective receptors (36).

To further investigate the therapeutic activity of Ex-DARP-FGF21 and FGF21, a control fusion protein, Ex-DARP without FGF21, was constructed. The Ex-DARP-FGF21 and Ex-DARP fusion proteins, soluble in *E. coli*, were purified using metal chelation affinity chromatography and size exclusion chromatography. These demonstrated high stability during purification without any obvious degradation in SDS-PAGE. An additional fusion protein, DARP-FGF21, served as a crucial control group for exploring the benefits of double agonists. However, difficulties were encountered during the protein preparation process, and pure DARP-FGF protein has not been obtained at this time. In a sandwich ELISA, Ex-DARP-FGF21 showed an EC50 of 4.5 nM in binding HSA, a value slightly higher than that of Ex-DARP. The half-life of Ex-DARP-FGF21 was extended to over 27 hours in mice, and both Ex-DARP-FGF21 and Ex-DARP maintained a glucose-lowering effect for over 3 days, significantly longer than that of exendin-4 or FGF21 alone (14, 41).

The potency of the dual agonist in vivo was evaluated by measuring the blood glucose levels and body weight of mice post-fusion protein treatment. Both Ex-DARP-FGF21 and Ex-DARP considerably reduced blood glucose levels in healthy and model mice. Conversely, the dual agonist targeting GLP-1R and FGF21R showed superior glucose-lowering efficacy compared to Ex-DARP in the OGTT and DIO mice. No significant difference was observed in the hypoglycemic effect between Ex-DARP-FGF21 and Ex-DARP in healthy mice due to the inadequate blood glucose fluctuation in normal mice to substantiate additive effects. Literature reveals that dual agonists of GLP-1 and FGF21, fused by ELP or immunoglobulin 1 (Fc), offer better blood glucose reduction, weight loss, and effect duration than GLP-1 or FGF21 monotherapy (39, 40). The ELP-fused protein can form a depot under the skin, releasing molecules steadily into circulation and resulting in an extended half-life of over 7 days. Also, GLP1-ELP-FGF21 had an inhibitory effect on weight gain, reducing body weight by up to 7.2 ± 2.3% (39). In contrast, more significant weight loss (above 10%) was observed with Ex-DARP-FGF21 treatment in our study using DIO model mice.

However, additional research is needed to enhance the study of these fusion proteins. The biological effects of Ex-DARP-FGF21 require further investigation to validate the designed dual agonist's synergistic effects on body weight control. Moreover, the linker peptide should undergo systematic evaluation and design to enhance the stability and solubility of the fusion protein for future applications.

### 5.1. Conclusions

In summary, we successfully designed a new long-acting dual agonist, exendin-4/FGF21, using albumin-binding DARPin as a carrier. We could express the Ex-DARP-FGF21 fusion protein in the *E. coli* system and purify it in a stable form with relative ease. In vitro, testing revealed a high binding affinity for HSA, and in vivo analysis demonstrated a long half-life of 27.6 hours. The synergistic effect of exendin-4 and FGF21 resulted in enhanced efficacy for blood glucose regulation and body weight control compared to exendin-4 alone. Given these promising results, we believe this dual agonist could emerge as a novel drug candidate for treating diabetes and obesity.

## Data Availability

The dataset presented in the study is available on request from the corresponding author during submission or after publication. The data are not publicly available due to further research on this drug in pharmacodynamic and toxicologic studies.
